# Pregnancy With SARS-CoV-2 Infection Complicated by Preeclampsia and Acute Fatty Liver of Pregnancy

**DOI:** 10.7759/cureus.15645

**Published:** 2021-06-14

**Authors:** Anisha Choudhary, Vinita Singh, Murari Bharadwaj, Archana Barik

**Affiliations:** 1 Obstetrics and Gynecology, Tata Main Hospital, Jamshedpur, IND; 2 Family Medicine, Tata Main Hospital, Jamshedpur, IND

**Keywords:** covid-19, pregnancy, preeclampsia, acute fatty liver of pregnancy, coronavirus

## Abstract

The coronavirus disease 2019 (COVID-19) pandemic has spread across the world in a relentless and merciless way. As the pandemic escalates, varied presentations and complications of the disease have been reported from all over the world. Pregnant women fall into a vulnerable group who have been reported to have more severe disease and need of intensive care when compared to non-pregnant women of the same age group. Preeclampsia is one of the most common co-morbidity seen in pregnant women with COVID-19 infection. Severe acute respiratory syndrome coronavirus 2 (SARS-CoV-2) infection can lead to worsening of pre-existing co-morbidities and extra vigilance is required in such cases. Here we present a case of a pregnant woman with COVID-19 infection with preeclampsia complicated by acute fatty liver of pregnancy and acute kidney injury. Although a rare diagnosis, a prompt multidisciplinary approach helped in achieving a favorable maternal and neonatal outcome.

## Introduction

The ongoing coronavirus disease 2019 (COVID-19) pandemic has spread across the globe in an alarming rate and is responsible for the death of more than two million people worldwide. Pregnancy affected with COVID-19 initially presented with uncertainties but with time more information is available about its effect on pregnancy outcome. Although respiratory symptoms are the most commonly observed clinical manifestation of the disease, a substantial number of infected patients may develop involvement of other systems and present with extra-respiratory manifestations like cardiac, gastrointestinal, hepatic, renal, neurological, ocular and hematological symptoms [1]. Liver function impairment is a common complication seen in patients with severe acute respiratory syndrome coronavirus 2 (SARS-CoV-2) infection and elevated levels of aspartate aminotransferase (AST) or alanine transaminase (ALT) are the most commonly seen manifestation of liver injury, reported in 14% to 53% of infected patients [2].

Preeclampsia in pregnancy can lead to grave complications such as hemolysis, elevated liver enzymes and low platelet (HELLP) syndrome, acute fatty liver of pregnancy (AFLP) and acute kidney injury. AFLP is rare, usually presents in the third trimester of pregnancy or early post-partum period. It is a potentially lethal complication for both the mother and fetus, with an approximate incidence of one in 7,000 to 16,000 pregnancies [3].

Recent data shows that pregnant women infected with SARS-CoV-2 are more likely to need intensive care treatment when compared to non-pregnant women of reproductive age [4]. Furthermore, it is seen that mothers with co-morbidities may be at increased risk of adverse outcomes, therefore it is important that emphasis is given on monitoring of such pregnancies. Here we report a case of twin pregnancy with COVID-19 complicated by preeclampsia and AFLP requiring intensive care with a favorable maternal and neonatal outcome.

## Case presentation

A 27-year-old primigravida with di-chorionic di-amniotic twin pregnancy was admitted at 35 weeks of gestation with pain abdomen for two hours. She gave history of low-grade fever for four days associated with mild cough. On admission she was icteric and afebrile, pulse rate was 88/minute, blood pressure was 142/94 mmHg, respiratory rate was 20/minute and oxygen saturation was 98% in room air. Prior to admission patient had regular antenatal visits, with no history of any medical disorder. Patient was admitted in COVID-19 suspect isolation ward and nasal swab was sent to test for COVID-19. Her ultrasound abdomen showed twin live fetus, first in cephalic and second in breech position, with normal liquor volume. Her urine dipstick test was done and showed +2 protein level. She was started on tablet labetalol 200 milligram twice daily. The routine blood investigations sent on admission were collected (Table 1) and liver function test was grossly deranged.

**Table 1:** Laboratory investigations of the patient on the day of admission. TLC: total leukocyte count; ALT: alanine transaminase; AST: aspartate aminotransferase; ALP: alkaline phosphatase; LDH: lactate dehydrogenase; GFR: glomerular filtration rate; CRP: C-reactive protein.

Parameter	On admission	Normal range
Hemoglobin (gm/dl)	10.6	11.5-16.5
TLC (cells per mm^3^)	7,300	4,000-11,000
Platelet (cells per mm^3^)	162,000	150,000-450,000
Prothrombin time (seconds)	11.5	11-16
International normalized ratio	1.07	0.8-1.2
Total bilirubin (mg/dl)	4.88	0.2-1
Direct bilirubin (mg/dl)	3.58	0.1-0.5
ALT (U/l)	473.2	5-40
AST (U/l)	728.5	5-45
ALP (U/l)	867.9	35-125
LDH (U/l)	596.9	208-378
Serum creatinine (mg/dl)	1.80	0.5-1.5
Estimated GFR (ml/min/1.73 m^2^)	44	60-120
Uric acid (mg/dl)	7.2	2.4-6
Serum sodium (mEq/l)	137	136-146
Serum potassium (mEq/l)	4.8	3.5-5.5
Serum chloride (mEq/l)	106	95-108
Serum ferritin (ng/ml)	140	20-120
CRP (mg/L)	22	0-5

Patient tested positive for COVID-19 (by rapid antigen test) and was shifted to the positive isolation ward. She tested negative for viral hepatitis, and an ultrasound upper abdomen was done which showed a normal study. As her platelet level was normal, provisional diagnosis of preeclampsia complicated by atypical HELLP syndrome and acute kidney injury was made. Decision of termination of pregnancy was taken and prophylactic magnesium sulfate was started for prevention of eclampsia. After two hours of admission, the patient had spontaneous rupture of membranes and thick meconium stained liquor. Her cervix was 2 cm dilated and 40% effaced. An emergency caesarean section was done under spinal anesthesia, twin babies were delivered weighing 2 kilogram (kg) and 2.1 kg, with APGAR (Appearance, Pulse, Grimace, Activity, and Respiration) score at birth of 8/10 each. Blood loss during the procedure was approximately 800 milliliters. All the healthcare professionals wore adequate personal protective equipment (PPE) during the procedure.

Both the babies were tested within 24 hours of delivery and reported negative for COVID-19 and were roomed in with the mother. Immediate post-operative period was uneventful. After 12 hours of caesarean section, patient complained of pain abdomen and vomiting and developed restlessness, confusion and breathlessness. On examination patient was disoriented, her pulse rate was 110/min, blood pressure was 130/90 mmHg, oxygen saturation in room air was 90% and blood sugar level was 47 mg/dl. Patient was started on oxygen inhalation and dextrose infusion. Emergency blood investigations were sent which showed worsening of bilirubin levels and deranged electrolyte levels (Table 2).

**Table 2:** Laboratory investigations of the patient on the days of illness. CRP: C- reactive protein; LDH: lactate dehydrogenase; TLC: total leukocyte count; ALT: alanine transaminase; AST: aspartate aminotransferase; ALP: alkaline phosphatase; GFR: glomerular filtration rate.

Parameter	Post-operative day 1	Post-operative day 2	Post-operative day 3	Post-operative day 4	Post-operative day 5	Post-operative day 6	Normal range
Hemoglobin (gm/dl)	9.5	-	10.7	10.9	10.8	-	11.5-16.5
TLC (cells per mm^3^)	17,200	-	14,300	12,900	11,600	-	4,000-11,000
Platelet (cells per mm^3^)	89,000	-	101,000	-	193,000	-	150,000-450,000
Prothrombin time (seconds)	14.5	-	12.9	9.2	-	-	11-16
International normalized ratio	1.35	-	1.19	0.87	-	-	0.8-1.2
Total bilirubin (mg/dl)	7.01	9.27	9.38	4.96	2.88	1.82	0.2-1
Direct bilirubin (mg/dl)	5.41	6.47	6.55	3.24	1.89	1.04	0.1-0.5
ALT (U/l)	232.6	145.9	114.9	44.9	47.7	33.0	5-40
AST (U/l)	274.2	158.0	111.5	56.0	48.1	27.0	5-45
ALP (U/l)	684.2	583.0	501.8	336.0	390.4	284.0	35-125
LDH (U/l)	656.0	-	398.3	-	-	300.2	208-378
Serum creatinine (mg/dl)	1.9	-	1.52	0.70	-	0.53	0.5-1.5
Estimated GFR (mL/min/1.73 m^2^)	37	-	46	118	-	-	60-120
Blood urea (mg/dl)	45.6	-	38.8	-	21	-	15-40
Uric acid (mg/dl)	8.7	-	-	-	-	-	2.4-6
Serum sodium (mEq/l)	117	125	-	135	-	-	136-146
Serum potassium (mEq/l)	3.3	4.5	-	4.3	-	-	3.5-5.5
Serum chloride (mEq/l)	100	107	-	109	-	-	95-108
Serum ferritin (ng/ml)	140	-	110	-	-	80	20-120
CRP (mg/L)	30	-	15	-	7.2	2.5	0 - 5
Total serum protein (gm/dl)	5.0	-	-	4.6	-	-	6.6 – 8.3
Serum albumin (gm/dl)	2.18	-	-	1.93	-	-	3.5 – 5.2
Serum globulin (gm/dl)	2.82	-	-	2.67	-	-	2.5 – 3.5

Her chest X-ray was done which showed bilateral basal opacities. A provisional diagnosis of AFLP was made according to Swansea criteria [5]. As this patient was developing multisystem organ dysfunction, she was shifted to positive intensive care unit (ICU) for further management. As her total count had increased and oxygen saturation was decreasing, she was started on oxygen support to maintain saturation >94% and iv antibiotics (injection cefoperazone and sulbactam). She was also started on low dose injection dexamethasone, injection vitamin K and IV fluids. Her blood pressure was controlled with tablet labetalol 200 mg thrice daily. Her lab parameters were monitored daily during the ICU stay. Patient responded to the supportive management, her sensorium improved, her oxygen requirement slowly decreased over six days and her lab parameters also showed improvement. On seventh post-operative day, she was maintaining oxygen saturation >95% on room air and was shifted to the general isolation ward. Injection dexamethasone and injection vitamin K were stopped and as her total count had also decreased iv antibiotics were changed to oral antibiotics (tablet cefuroxime). She was discharged on post-operative day eight and on tablet cefuroxime (500 mg twice daily for three days), tablet labetalol (200 mg thrice daily), iron and calcium supplementation for one month. She was followed up two weeks later and her renal and liver function tests were normal.

## Discussion

Preeclampsia is one of the most commonly associated co-morbidity with COVID-19 infection in pregnancy [6]. As placental angiotensin-converting enzyme 2 (ACE2) is highly expressed at the maternal-fetal interface, its dysregulation by SARS-CoV-2 might be involved in the high rates of preeclampsia associated with severe and critical COVID-19 infected pregnant women [7]. Both preeclampsia and COVID-19 infection are microvascular diseases resulting in a prothrombotic state which may lead to a synergistic effect and severe clinical manifestation as seen in our patient.

AFLP is a rare life-threatening disease. Majority of women with AFLP present in the third trimester of pregnancy; however, there are isolated case reports with presentation in early post-partum period [8]. Potential risk factors for AFLP include nulliparity, multiple gestation, preeclampsia or HELLP syndrome, male fetus and prior episode of AFLP [9]. It is believed that preeclampsia, HELLP syndrome, thrombocytopenic purpura and AFLP may be a spectrum of the same illness. Clinical diagnosis of AFLP is challenging due to its non-specific symptoms and varying clinical findings. Its symptoms mimic that of HELLP syndrome, viral hepatitis and obstetric cholestasis and the best approach to diagnosis remains to rule out other causes. AFLP is associated with higher degrees of liver enzyme derangement and coagulation abnormalities when compared to HELLP syndrome. Our patient tested negative for viral hepatitis, she had no complains of pruritus and although she had preeclampsia, features of hypoglycemia, disorientation and bilirubin level more than five mg/dl are not commonly seen with HELLP syndrome unless associated with AFLP.

The Swansea criteria [5] for the diagnosis of AFLP was relevant in our patient as she satisfied more than six criteria as given in the table below (Table 3).

**Table 3:** Swansea criteria for the diagnosis of AFLP [5]. Criteria fulfilled – vomiting, abdominal pain, elevated bilirubin, hypoglycemia, elevated transaminases, elevated urea and creatinine, encephalopathy, coagulopathy ALT: alanine transaminase; AST: aspartate aminotransferase; APTT: activated partial thromboplastin time; AFLP: acute fatty liver of pregnancy.

Swansea criteria
Vomiting
Abdominal pain
Polydipsia/polyuria
Encephalopathy
Elevated bilirubin > 14 µmol/l
Hypoglycemia < 4 mmol/l
Elevated urea > 340 µmol/l
Leukocytosis > 11 x 10⁹ /l
Ascites or bright liver on ultrasound scan
Elevated transaminases (AST or ALT) > 42 IU/l
Elevated ammonia > 47 µmol/l
Renal impairment: creatinine > 150 µmol/l
Coagulopathy: prothrombin time > 14 seconds or APPT > 34 seconds
Micro-vesicular steatosis on liver biopsy

Hepatic dysfunction has been commonly associated with COVID-19 infection [10]. Deng et al. [11] reported a 29.7% prevalence of liver injury in pregnant patients infected with COVID-19, and that these patients had worse inflammation than those without liver injury. Several factors have been associated with elevation of serum aminotransferases and acute liver damage in COVID-19 infected patients. These include severe hypoxemia due to acute respiratory failure, drug interactions, septic shock and multiorgan dysfunction [12]. At present there is insufficient evidence for direct SARS-CoV-2 virus-related hepatocyte injury; however, liver dysfunction has been continuingly related to severe COVID-19 infection and intensive care admission [13,14].

Morton [15] had reported a case of influenza hepatitis followed by AFLP and described the possible mechanism linking the two disease processes as impaired hepatic fatty acid oxidation mediated by the release of cytokines by Kupffer cells during infection with the influenza virus. It is possible that SARS-CoV-2 activated a similar response in this patient resulting in a synergistic effect on the existing preeclampsia leading to her worsening and multiorgan involvement.

## Conclusions

This case illustrates the complexities of COVID-19 in pregnancy, the overlap of symptoms and the increased severity of associated co-morbidities. As COVID-19 in pregnancy predisposes to hepatic dysfunction, health care workers should be extra vigilant in cases of pregnancy with preeclampsia or existing hepatic disorders, as the synergistic effect may lead to worsening of the patient, AFLP and multiorgan dysfunction.
